# The current state of validated small molecules inhibiting SARS-CoV-2 nonstructural proteins

**DOI:** 10.3906/biy-2106-42

**Published:** 2021-08-30

**Authors:** Fatih KOCABAŞ, Merve USLU

**Affiliations:** 1 Department of Genetics and Bioengineering, Faculty of Engineering, Yeditepe University, İstanbul Turkey

**Keywords:** SARS-CoV-2, COVID-19, small molecules, NSPs

## Abstract

The current COVID-19 outbreak has had a profound influence on public health and daily life. Despite all restrictions and vaccination programs, COVID-19 still can lead to fatality due to a lack of COVID-19-specific treatments. A number of studies have demonstrated the feasibility to develop therapeutics by targeting underlying components of the viral proteome. Here we reviewed recently developed and validated small molecule inhibitors of SARS-CoV-2’s nonstructural proteins. We described the validation level of identified compounds specific for SARS-CoV-2 in the presence of in vitro and in vivo supporting data. The mechanisms of pharmacological activity, as well as approaches for developing improved SARS-CoV-2 NSP inhibitors have been emphasized.

## 1. Introduction

Coronaviruses (CoVs) are enveloped positive-stranded RNA viruses that typically result in respiratory infections (Alam et al., 2020). Coronaviruses infections were considered relatively benign to humans before the severe acute respiratory syndrome (SARS) (Taştan et al., 2020). As of August 2021, an estimated 198 million people have been infected worldwide by the severe acute respiratory syndrome coronavirus 2 (SARS-CoV-2) and caused over 4.2 million deaths (World Health Organization, 2021). There are no specific therapies against COVID-19, which urges the identification of potential therapeutic targets and the development of COVID-19 therapies.

SARS-CoV-2 is a member of the β-coronavirus family that possesses the large Spike proteins on the surface of the virus that forms their crown-like shape. The viral genome consists of up to 32,000 bases (Jena et al., 2021). The genomic RNA consists of a variable number of open reading frames (ORFs) (Jena et al., 2021). It has been stated that the viral genome encodes approximately 29 proteins composed of 4 structural proteins and 25 nonstructural proteins (NSPs) and accessory proteins (Hasöksüz et al., 2020; Güler et al., 2021; Jena et al., 2021). ORF 1ab of SARS-CoV-2 encodes NSPs and takes part in the translation of two polypeptides known as pp1a and pp1ab. The nonstructural protein pp1a comprises NSP1 to NSP11 and pp1ab corresponds to NSP12 to NSP16 (Hasöksüz et al., 2020). The 10,000kb region through 3’end encodes structural proteins named membrane (M), envelope (E), surface (S), and nucleocapsid (N). The SARS-CoV-2 viruses are enveloped, spherical and their size is about 80–120 nm in diameter (Petrosillo et al., 2020; Güler et al., 2021). Due to the many outward homotrimeric glycosylated S protein projections, their appearance resembles a solar corona (Bulut and Kato 2020). The capsid contains the N protein and is further encircled with a membrane including the M protein and the E protein that are participating in virus budding, and the spike glycoprotein, which is important for binding the host receptor following membrane fusion and the entry of virus in the host cells. Along with these four structural proteins, nine accessory proteins ORF3a, ORF3b, ORF6, ORF7a, ORF7b, ORF8, ORF9b, ORF9c, and ORF10 are encoded at the 3’ end as well (Güler et al., 2021). Accessory proteins were considered to participate in the virulence as well as the host-virus interactions. 

SARS-CoV-2’s spike protein binds to its cognate host cell receptor, which is angiotensin-converting enzyme 2 (ACE2) (Hasöksüz et al., 2020). After the fusion inside the host cells, the viral genome is delivered as a single-stranded RNA, which is replicated by RNA-dependent RNA polymerase (RdRp) protein to generate viral genomes. Thereafter, the translation of viral polyproteins occurs with the help of human translation machinery. Upon this cellular entry, the ORF-1a and ORF-1ab have been subjected to the translation and hence the formation of pp1a and pp1ab polyproteins. These large nonstructural proteins are cleaved into smaller 16 nonstructural proteins by the viral main protease (Mpro) and papain-like protease (PLpro). This is followed by the molecular assembly and the formation of functional viral polymerase, which is also known as viral replicase. Assembly of several structural proteins and encapsulation of genomic RNA end up with plenty of viral progenies and eventually exit from the host cell consequently spreading an infection to the other part of the body. As the virus continues to replicate, it hits the lungs that result in inflammation and pneumonia (Azkur, et al. 2020; Bulut and Kato 2020; Hoffmann, et al. 2021).

The NSP1 and NSP3 take a role in the suppression of interferon signaling and hindering host immune response via fastening cellular degradation and hampering RNA translation in the host (Ysrafil, et al. 2020). The NSP2 has been shown to reduce interferon production and repressing antiviral responses. The NSP4 and NSP6 participate in the construction of the transmembrane scaffold protein, especially forming double-membrane vesicles. Binary complexes of NSP7 and NSP8 facilitate the viral RNA processing via NSP12 (Ysrafil et al., 2020). While NSP9 contributes RNA binding protein phosphatase, NSP10, NSP16,and NSP12 induce exonuclease (ExoN) and 2′-O-methyltransferase (2′-O-MTase) activity (Ysrafil et al., 2020). NSP12 plays a role in RNA synthesis as an RNA-dependent RNA-polymerase. The NSP13 as an RNA helicase holds nucleoside triphosphatase (NTPase) activities (Ysrafil et al., 2020). NSP14 takes a role in viral genome proofreading (Ysrafil et al., 2020). NSP15 participates in the activities of viral endoribonuclease and viral protease (Ysrafil et al., 2020). The NSP16 manages the repression of melanoma differentiation-associated protein 5 (MDA5) and interference of the innate immunity regulation (Ysrafil et al., 2020). Various studies addressed the possibilities to target viral NSPs to develop COVID-19 specific therapeutics. 

## 2. NSPs of SARS-CoV-2 as drug targets

### 2.1. NSP1: the host translation inhibitor protein of SARS-CoV-2

SARS-CoV-2 NSP1 viral protein functions as a ribosome modulator, preventing host translation and promoting SARS-CoV-2 protein production (Tidu et al., 2021). NSP1 binds to the host ribosomes and inhibits the mRNA entry, making it one of the first proteins generated after viral infection, thus cellular translation is inhibited. Virus translation continues despite the presence of NSP1 on the ribosome. The cis-acting RNA hairpin SL1 in the 5’UTR of SARS-CoV-2 is recently shown to be the underlying molecular mechanism of viral evasion of NSP1 suppression (Tidu et al., 2021).

In the late stages of the infection, SL1 is involved in the translation of viral RNAs. As a result, treatment techniques that target SL1 could have an impact on viral translation in both the early and late stages of infection. It was previously recommended that coronavirus NSP1 proteins be targeted for the development of innovative treatment techniques (Tidu et al., 2021). Because SL1 is found in SARS-CoV-2 viral transcripts, targeting viral translation may be possible once small molecule or other targeting approaches are developed.

Structure analysis of SARS-CoV-2 NSP1 demonstrates a seven-stranded β-barrel with α-helix on one side (Clark et al., 2021). Comparing the structure and sequence of SARS-CoV-2 NSP1 with SARS-CoV NSP1 shows high similarity. However, there are a few discrepancies between the two proteins that might affect how they operate in their distinct viral life cycles. Because of the enhanced polar interactions between the amino acids that make up the loops and the globular domain, some of the major loops are in alternate conformations. Changes in the electrostatic surface potential between the two proteins are due to amino acid variations. Secondary structural features, such as an extra β-strand (β5) and 3_10_ helices, are present in SARS-CoV-2 NSP1 only. Given the importance of NSP1 in the SARS-CoV-2 virulence, these variations might explain the higher virulence of SARS-CoV-2. Besides these important features, there is still further research needed to develop small molecule inhibitors that are unique to SARS-CoV-2 NSP1.

### 2.2. NSP2: SARS-CoV-2 the host signaling and mitochondrial biogenesis modulator

The NSP2 protein of SARS-CoV-2 is considered to have a function in altering the host cell environment. NSP2 protein binds to host proteins PHB1 and PHB2, which are involved in mitochondrial biogenesis and signaling (Cornillez-Ty et al., 2009). The SARS-CoV and SARS-CoV-2 NSP2 proteins share 68.3% of sequence homology (Yoshimoto, 2020). Gupta et al. (2021) recently reported the structure of SARS-CoV-2 NSP2 and showed that NSP2 has several contact surfaces for host proteins (Gupta et al., 2021). These investigations provided information on how to target these viral proteins. More study has to be done, however, to develop SARS-CoV-2 specific small molecule inhibitors of NSP2.

### 2.3. NSP3: SARS-CoV-2, a large multidomain and multifunctional protein

SARS-CoV NSP3 protein is a large multidomain protein that includes UBL-1, AC, ADRP, SUD, UBL-2, PLpro, NAB, G2M, TM1-2-3-4, ZN, and Y1-2-3 domains (reviewed in (Báez-Santos et al., 2015)). It is believed that NSP3 plays a major role in viral replication as a central scaffold protein of the replicase complex. Deubiquitinating/DeISGylating domain of NSP3 is also known as PLpro domain, which recognizes and cleaves at LXGG amino acids between NSP1-2, NSP2-3, and NSP3-4, at C-terminus of human ubiquitin (UB) and interferon-stimulated gene 15 (ISG15) (Figure). This cleavage is essential for viral replication. SARS-CoV NSP1 protein is involved in the host mRNA degradation and inhibition of the host mRNA translation (Reviewed in (Báez-Santos et al., 2015)). This promotes the viral gene expression and downregulation of the innate immune response gene expression. SARS-CoV NSP3 interacts with NSP4 and NSP6. Following this, NSP6 is involved in the inhibition of autophagosome activity and delivery of viral components to lysosomes for degradation (reviewed in (Báez-Santos et al., 2015)). NSP3 is associated with the reduction of proinflammatory cytokines and chemokines endogenously. In addition, it has been shown to upregulate CCL5, IFN-β, and CXCL10 in the cells. SARS-CoV NSP3 protein antagonizes the innate immune induction of type I interferon by blocking the phosphorylation, dimerization, and subsequent nuclear translocation of the host IRF3 (Ratia et al., 2006) and prevent the host NF-κB signaling thus cellular antiviral response (Ratia et al., 2006; Kocabas and Aslan, 2015; Kocabaş and Ergin, 2016).

Comparison of SARS-CoV PLpro and SARS-CoV-2 PLpro shows 83% sequence identity with differential substrate preference (McClain and Vabret, 2020; Shin et al., 2020). Shin et al. (2020) showed the deISGylation activity of SARS-CoV-2 PLpro and its involvement in the etiology of COVID-19. It appears that inhibiting PLpro not only prevents viral replication but also restores antiviral immunity, making PLpro a particularly intriguing target for future therapeutic development (reviewed in (Zhu et al., 2021)). While SARS-CoV-2 PLpro prefers to cleave the ISG15, SARS-CoV PLpro prefers to break ubiquitin chains (Shin et al., 2020). In addition, SARS-CoV-2 PLpro helps in the cleavage of ISG15 from IRF3 and inhibits type I interferon responses following infection.

Klem et al (2020) have tested SARS-CoV PLpro inhibitors rac3j, rac3l, and rac5c against SARS-CoV-2 PLpro (Klemm et al., 2020). In addition, rac3j, rac3l, and rac5c partially blocked SARS-CoV-2 infection in Vero E6 cell-based assay. Kuo et al. (2021) also showed in vitro inhibition of both Mpro and PLpro with levothyroxine and manidipine-2HCl, which are FDA-approved medicine (Kuo et al., 2021). 

It has been recently shown that inhibition of SARS-CoV-2 PLpro with GRL-0617, a naphthalene-based inhibitor, lowers viral replication in infected cells while maintaining the antiviral interferon pathway and impairing the virus’s cytopathogenic impact (Freitas et al., 2020; McClain and Vabret, 2020). A recent study further docked GRL-0617, 3k, hypericin, cyanidin-3-O-glucoside, and rutin into the inhibition pocket of SARS-CoV-2 PLpro and validated their inhibitory effect in vitro (Pitsillou et al., 2020). PLpro deubiquitinase (DUB) enzymatic assay showed an IC50 value of 1.7 μM for GRL-0617 against SARS-CoV-2 PLpro. Hypericin inhibited 90%, rutin 50%, and cyanidin-3-O-glucoside 42% of DUB activity of SARS-CoV-2 -PLpro at 100 μM dose.

Osipiuk et al (2021) studied the structure of SARS-CoV-2 PLpro along with the identification and validation of seven inhibitors (Osipiuk et al., 2021). Five of these were also validated to inhibit viral replication in cell culture-based assays. Small molecule 1 (GRL0617) inhibited SARS-CoV-2 PLpro with an IC50 value of 2.3 μM. Small molecules 2, 3, 4, 5, 6, and 7, which have IC50 values ranging from 5.1 to 32.8 μM, are derivatives of GRL0617. Small molecules 1, 4, and 7 have EC50 values ranging from 1.4 to 5.2 μM. Small molecules 2 and 3 failed to inhibit viral replication. These findings suggest that additional improvement is conceivable, particularly in the case of small molecule 5, which is a poor binder but a potent viral inhibitor.

SARS-CoV NSP3 protein has not only include the PLpro domain but also the AC, ADRP, SUD, UBL-2, NAB, G2M, TM1-2-3-4, ZN, and Y1-2-3 domains, which require further studies and knowledge to design and develop targeted treatments.

**Figure:**
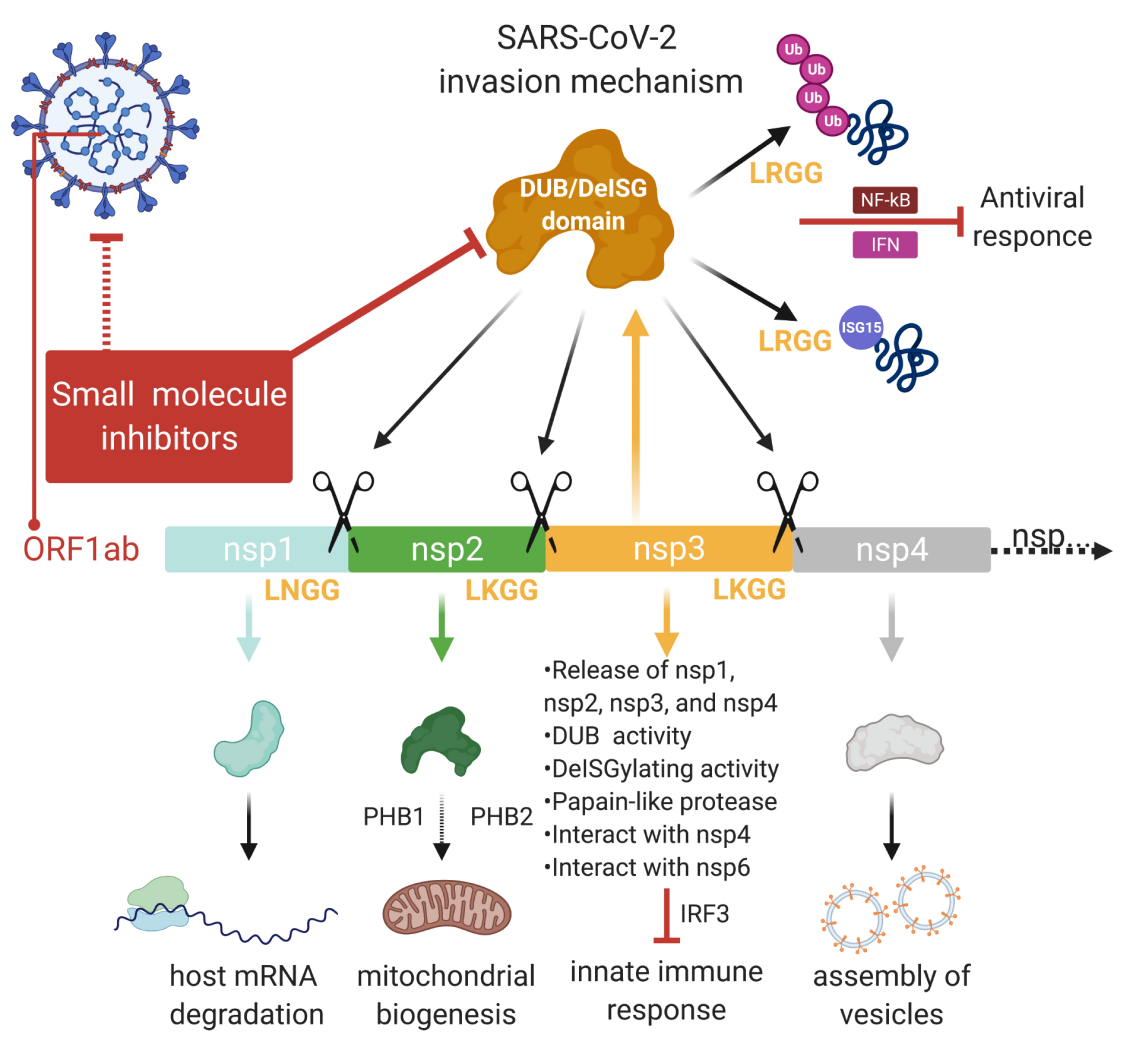
Viral invasion mechanism and role of SARS-CoV-2 NSP3 protein. NSP3 protein is involved in processing of NSP1-4 proteins via cleavage at LNGG sites. This leads to formation of functional NSP1 (involved in host mRNA degradation), NSP2 (involved in metabolic modulation of host cell via PHB1 and PHB2), and NSP4 (involved in assembly of viral vesicles). NSP3 with its various domains involves DUB, DeISGlating activity, and interact with NSP4 and NSP6 to modulate innate immune response.

### 2.4. NSP4: SARS-CoV-2 membrane remodeling and viral vesicle assembly factor

The majority of +stranded RNA virus infection stimulates the readjustment of host cell membranes that harbors the formation of a viral replication complex known as the replication and transcription complex (RTC) (Den Boon and Ahlquist, 2010; Matsuyama, et al., 2020). This membrane remodeling following RTC formation post virus infection are the important sites for the viral RNA synthesis and provide protection of newly synthesized RNA from innate immune system components in the host (Den Boon and Ahlquist, 2010). SARS-CoV infection has been shown to induce the replication-associated membrane structures which contain convoluted membranes (CMs) and double-membrane vesicles (DMVs) as two interconnected membrane structures (Goldsmith et al., 2004). Among all coronavirus NSPs, the multiple transmembrane domains have been established by NSP3, NSP4, and NSP6. Another study showed the cellular localization of SARS-CoV NSP3, NSP4, and NSP6 that induce DMVs formation in the SARS-CoV infected cells through the confocal and electron microscopy analysis (Angelini et al., 2013). It has been intriguingly shown that the suppression of ER export machinery by using the H89 kinase inhibitor or by expression of a mutant Sar1[H79G] inhibited the coronavirus replication (Hagemeijer et al., 2012). This highlighted the significance of the early secretory pathway in the RTC formation.

Another study conducted with PLA assay and immunoprecipitation demonstrated the importance of the key residues in the membrane rearrangement and the NSP3 co-expression (Sakai et al., 2017). The NSP4 residues between 112 and 164 are the sites where bind to NSP3. H120 and F121 residues in NSP4 have been identified as conserved residues between beta coronaviruses (Sakai et al., 2017). These residues participate in viral RNA replication with the interaction of NSP3 and employing membrane remodeling. Mutations of H120N and F121L resulted in the disruption of viral RNA replication through the damage in the membrane re-arrangements. For this reason, inhibition of NSP4 interaction with other proteins might be utilized as a target for the development of therapeutics. Further study, however, is required to develop small molecules targeted to SARS-CoV-2 NSP4.

### 2.5. NSP5: SARS-CoV-2 main protease (Mpro or 3CLpro)

The ORF1ab encodes the Mpro, which cleaves pp1a and pp1ab polyproteins into 16 nonstructural proteins (Gordon et al., 2020; Xie and Chen, 2020). Studies revealed structural similarities and inhibitor binding modes in MERS-CoV, SARS-CoV, and SARS-CoV-2 main proteases (Mali, 2020; Ullrich and Nitsche, 2020). The viral protease enzymes are vital for viral protein maturation in various viruses because it allows the processing of proproteins after they are translated into the host cell’s cytoplasm. Thus, viral proteases are routinely considered as potent drug targets to develop potential therapeutics. Blocking or reduction of the mature viral particle generation can be achieved by inhibiting viral proteases. To this end, many antiviral medications that target proteases such as HIV-1 protease and HCV protease have been developed and approved by FDA (Wang et al., 2015). As a result, developing antiviral medicines that inhibit SARS-CoV-2 Mpro might be useful in the clinic (reviewed in (Mengist et al., 2021)). 

Several small molecules exhibited efficient binding and inhibition of SARS-CoV-2 Mpro as they were validated by in vitro assays, antiviral activity towards SARS-CoV-2, and in vivo infected mice investigations (Rathnayake et al., 2020; Riva, et al., 2020). The structure of SARS-CoV-2 Mpro in association with various potential small molecule inhibitors such as peptidomimetic α-ketoamide inhibitors was also recently shown (Zhang et al., 2020). Dai et al (2020) developed aldehyde-based small molecules 11a and 11b that inhibited SARSCoV-2 Mpro up to 100 percent at 1 μM (Dai et al., 2020). These two small molecules were also shown to prevent SARS-CoV-2 infection (Dai et al., 2020). The scientists improved a previously proposed inhibitor (11r to 13a), which is resulting in longer plasma stability (Mengist et al., 2020; Zhang et al., 2020). Zhang et al. (2020) reported compounds 11r and 13a having IC50 values of 0.18 μM and 2.39 for SARS-CoV-2 Mpro, respectively (Zhan, et al., 2020). Small molecules 13a and 13b showed selective binding to SARSCoV-2 Mpro, reduction of enzyme activity, and viral infectivity. Compound 13b had an IC50 value of 0.67 μM against SARS-CoV-2 Mpro and an EC50 value of around 5 μM when tested in vitro with SARS-CoV-2 assays in human Calu3 cells (reviewed in (Sharma et al., 2020)). 

N3 interacted with several hydrogen bonds and acted as an irreversible Michael acceptor inhibitor of SARS-CoV-2 Mpro (Griffin, 2020; Jin et al., 2020). Another research determined the structure of N3 binding into the SARS-CoV-2 Mpro substrate-binding pocket (Jin, et al. 2020). Infected Vero cells with SARS-CoV-2 demonstrated high antiviral activity of N3 at 10 μM. Jin et al. (2020) also revealed that carmofur binds to the SARS-CoV-2 Mpro Cys145 residue and inhibits viral replication (Jin et al., 2020). Ebselen, an antiinflammatory and antioxidant, has also been shown to bind to the active site of SARS-CoV-2 Mpro and has significant antiviral activity, thus, it has been suggested to be useful in treating COVID-19 (Jin et al., 2020; Sies and Parnham, 2020).

Su et al. (2020) identified natural molecules originating from Chinese traditional medicine, baicalin, and baicalein as new inhibitors of the SARS-CoV-2 Mpro with antiviral activity in Vero E6 cells, with IC50 values of 6.4 and 0.9 μM respectively (Su et al., 2020). They demonstrated that baicalein can inhibit SARS-CoV-2 Mpro through hydrogen bonds between baicalein and Leu141/Gly143 and Ser144/His163 residues (Su et al., 2020).

Vuong et al. (2020) utilized a prodrug approach to develop an SARS-CoV-2 Mpro inhibitor. They have shown conversion of GC376 after forming a covalent bond with Cys145 into GC373 in SARS-CoV-2 Mpro (Vuong et al., 2021; Vuong et al., 2020). Additional hydrogen bonds were also reported between SARS-CoV-2 Mpro catalytic region and GC373 indicating a high binding affinity and inhibition as low as nano-molar level between enzyme and ligand (Liu et al., 2021; Sharun et al., 2021). Iketani et al. (2021) have reported molecules namely compound 4, GC376, and MAC-5576 as inhibitors of SARS-CoV-2 main protease (Iketani et al., 2021). Compound 4 and GC376 showed IC50 values of 151 nM and 160 nM against SARS-CoV-2 Mpro, respectively. Further studies showed that compound 4 and GC376 could inhibit SARS-CoV-2 viral replication in vitro at EC50 values of 2.8 μM and 2.1 μM, respectively. While MAC-5576 had the lowest IC50 value of 81 nM against SARS-CoV-2 Mpro, it was not capable of inhibiting SARS-CoV-2 viral replication. Ma et al. (2020) also showed that GC-376 along with boceprevir, and calpain inhibitors II and XII as potent Mpro inhibitors with antiviral activity (EC50 as low as 0.49 μM) (Ma et al., 2020).

Riva et al. (2020) performed a drug repurposing screening to identify potent SARS-CoV-2 inhibitors (Riva et al., 2020). Intriguingly, several cysteine protease inhibitors MDL-28170, Z LVG CHN2, VBY-825, and ONO 5334 were shown to pose antiviral activity suggesting that targeting viral proteases is a plausible strategy to develop COVID-19 therapeutics. Rathnayake et al (2020) studied 6e and 7j in SARS-CoV-2 infected Vero E6 cells to demonstrate its antiviral activity (Rathnayake et al., 2020). The scientists also demonstrated that these Mpro inhibitors can enhance the survival of coronavirus-infected mice (Rathnayake et al., 2020). 

Coelho et al. (2020) employed screening to find possible SARS-CoV-2 Mpro inhibitors (Coelho et al., 2020). The study allowed identifying and validating thirteen potential inhibitors of recombinant SARS-CoV-2 Mpro with IC50 values ranging from 0.2–23 μM. Thimerosal, phenylmercuric acetate, and Evans blue were the most potent inhibitors with IC50 values of less than 1 μM. Glaab et al. (2021) selected 95 small molecules after in silico studies to determine in vitro SARS-CoV-2 Mpro inhibitory activity (Glaab et al., 2021). Two compounds rottlerin and M-8524 (CID 46897844) showed IC50 values of 37 μM and 31 μM, respectively. 

Qiao et al. (2021) reported 32 new bicycloproline based SARS-CoV-2 Mpro inhibitors with IC50 values ranging 7.6 to 748.5 nM (Qiao, et al. 2021). Further studies with compounds MI-09 (EC50 = 0.86–1.2 μM) and MI-30 (EC50 = 0.54–1.1uM) showed robust antiviral activity in vitro and in vivo. In addition, MI-09 and MI-30 reduced lung viral load and lesions in a SARS-CoV-2 infected hACE2 transgenic animal model. These findings suggest that administering MI-09 or MI-30 may effectively limit SARS-CoV-2 replication and mitigate SARS-CoV-2 induced lung lesions, which are establishing a major step in the development of anti–SARS-CoV-2 medicines.

Previously identified HCV NS3/4A protease inhibitors have been proposed to bind to SARS-CoV-2 Mpro with an antiviral activity (reviewed in (Mengist et al., 2021)). A number of ligand-SARS-CoV-2 Mpro structure analyses reported different compounds including N3 (PDB:6LU7), 11a (PDB:6LZE), 11b(PDB:6M0K), X77 (PDB:6W63), 13b (PDB:6Y2G), Baicalein (PDB:6M2N), Boceprevir (PDB: 7K40), Narlaprevir (PDB:7JYC), Telaprevir (PDB:7K6D), Carmofur (PDB:7BUY), GC373 (PDB:6WTK), GC376 (PDB:6WTT), biotin-PEG(4)-Abu-Tle-Leu-Gln-vinylsulfone (PDB:6Z2E), x0397 (PDB:5RGI), X2754 (PDB:5RHF), and X2705 (PDB: 5RH7). 

In short, these small molecule inhibitors blocking the activity of NSP5 showed that targeting viral main protease is a plausible strategy to achieve the development of SARS-CoV-2 specific therapeutics. 

### 2.6. NSP6: SARS-CoV-2 autophagosome modulator

Several NSPs are collected into replication complexes on the ER cytoplasmic region. Assembly of replicase proteins and genome replication are concurrently associated with the formation of DMVs and autophagy activation. Although autophagy machinery-mediated degradation can promote the innate defense mechanism against virus infection, autophagosomes of (+) strand RNA viruses can also provoke the infection by enabling the replicase proteins assembly (Cottam et al., 2014). As a cellular response, autophagy constitutes autophagosomes that transport long-lived proteins and damaged cellular proteins to lysosomes for their degradation. This also supplies a short-term obtainment of amino acids when starvation happens (Wang and Klionsky, 2003). Autophagy with this potential to degrade the massive quantities of cytoplasm enables host cells to fight intracellular pathogens and provides the presentation of invader components to the immune system (Levine, 2005).

The protein NSP6 of infectious bronchitis virus, mouse hepatitis virus, avian coronavirus, and SARS has been shown to induce autophagy. Since it has multiple membrane-spanning domains and located in the ER, NSP4 produces autophagosomes from ER (Cottam et al., 2011). A study conducted with viral NSP6 has been also shown that NSP6 coronavirus protein restricts the expansion of autophagosomes whether generated directly via NSP6 protein or indirectly in response to starvation or small molecule Torin1 mediated inhibition of mTOR pathway signaling (Cottam et al., 2014). It is unclear why coronaviruses restrict the expansion of autophagosomes even if the infection with coronavirus has been stated to activate autophagy. More work is needed for the NSP3, NSP4, and NSP6 sub-complex interaction in the formation of DMVs as well as the cooperation with autophagy machinery. Targeting autophagy or the mTOR signaling pathway by using small molecules could provide more understanding for the design of viral therapeutics as well as broadening the reservoir of antivirals. Studies also suggest that SARS-CoV-2 NSP6 could be directly targeted by small molecules (Pandey, et al. 2020). Analysis of haloperidol and dextromethorphan, especially, shows that they bind tightly to NSP6 and cause alterations in its structure and dynamics relevant to their distinct modes of action. However, further studies are still needed for the discovery and development of SARS-CoV-2 NSP6 specific inhibitors. 

### 2.7. NSP7, NSP8, and NSP12: SARS-CoV-2 RNA-dependent RNA polymerase complex

The genome of (+) stranded RNA viruses possesses RNA-dependent RNA polymerase (RdRp) to carry out viral genome replication (Paul and Wimmer, 2015). Upon the infection into host cells, the replication of viral RNA cannot launch until the polymerase is translated directly from genomic RNA of (+) stranded RNA viruses. Therefore, the activity of RdRp plays a critical role in the synthesis of SARS-CoV-2 RNA. NSP7-NSP8 complex has been stated to enhance the RNA polymerase activity in a primer-dependent manner (Te Velthuis et al,. 2012). In addition to NSP12 protein as a presumed main RdRp, the polymerase activity assays and NSP8 mutagenesis studies suggested this unique NSP7 and NSP8 complex is most likely to be another SARS-CoV polymerase that boosts the RdRp activity (Te Velthuis, et al. 2012). 

The NSP8 protein has been reported in the activity of oligonucleotide synthesis in a template-dependent manner (Imbert et al., 2006). This protein binds to a specific short sequence in the ssRNA genome of coronavirus and is able to perform de novo RNA synthesis up to six nucleotides. Subsequently, this can be utilized as a primer for the RdRp synthesis with low fidelity indicating the similarity with DNA-dependent RNA primase (Imbert et al., 2006). CoVs exhibit this specialized NSP8 RNA enzyme activity for the primer synthesis known as primase. It has been stated that NSP8 protein could be an interesting target against CoVs and inhibition of NSP8 was determined by using GTP analogs including 3’-dGTP, ddGTP, and 20-O-methyl-GTP (Imbert et al., 2006). While 3’-dGTP showed efficient inhibition of NSP8 RdRp, ddGTP and 20-O-methyl-GTP were found as weak inhibitors. Alanine-scanning mutagenesis indicated that RdRp activity of NSP8 was aborted or seriously diminished with K58A, R75A, K82A, and S85A mutations. 

Analysis of replication complex including NSP7 and NSP8 showed that SARS-CoV-2 NSP8 undergoes conformational changes and interacts with the departing RNA. A model for the transition from primase to polymerase complex sheds light on the mechanism of remdesivir incorporation and delayed chain termination in viral RNA replication (Wang, et al. 2020). In addition, the GST-pull down assays indicated the interaction of NSP8 with NSP12 (Imbert et al., 2006). Thus, NSP8/NSP12 interaction was also proposed to be targeted to develop inhibitors of this complex, which is important in viral replication (Mutlu et al., 2020). The NSP12–NSP8 complex of SARS-CoV-2 was targeted utilizing a structure-based and computer-aided drug design strategy (Mutlu et al., 2020). Despite the partial mobility of the RX-3117 and Nebivolol ligands in the binding cavity, they were not dislodged from the binding pocket after 100 nanoseconds suggesting that they could potentially inhibit NSP12–NSP8 complex of SARS-CoV-2 and could be further validated in order to pave the way for new COVID-19 treatment approaches. These studies provided an insight into how to target these viral proteins. Further research is needed, however, to develop SARS-CoV-2 specific NSP7 and NSP8 small molecule inhibitors.

RdRp is a key catalytic component for RNA synthesizing machinery. In all (+) strand RNA viruses, RdRp produces negative-strand RNA, new genome elements, and mostly subgenomic messenger RNAs. In coronaviruses, RdRp catalyzes the viral RNA synthesis and produces negative-strand RNA starting from 3’-poly-A-tail by using (+) stranded RNA strand as a template. In order to initiate the genomic RNA synthesis, the viral RdRps operate two essentially different mechanisms which are de novo known as primer independent RNA synthesis and primer dependent RNA synthesis (Van Dijk et al., 2004). During de novo synthesis, the first nucleotide 3’hydroxyl group acts as a primer, and the second nucleotide 5’-phosphate group is added through the formation of phosphodiester bonds. In the case of primer-dependent RNA synthesis, the initiation of new RNA strand synthesis depends on either an oligonucleotide or a protein primer. SARS-CoV NSP12 acts as a canonical RdRp that catalyzes the viral RNA synthesis and plays a crucial role in the viral replication and transcription with the assistance of SARS-CoV NSP7 and NSP8 (Subissi et al., 2014a; Subissi et al., 2014b). Thus, the SARS-CoV-2 polymerase complex is constituted with NSP12 RdRp and NSP7/NSP8 cofactors (Gao et al., 2020; Peng et al., 2020). It has been reported that the activity of SARS-CoV NSP12 is highly augmented upon the binding of the NSP7/NSP8 complex (Subissi et al., 2014a; Subissi et al., 2014b). 

The development of in vitro RdRP assays, which allow screening and identification of small molecules, are crucial for therapeutics targeting viral RNA synthesis (Kocabas et al., 2015). Various methods have been proposed and reported to function in the identification of various virus RdRp inhibitors. Nucleotide analogs that target viral RNA polymerase have been shown to be a promising antiviral medication (Lu et al., 2020). Lu et al. (2020) developed a nonradioactive primer extension assay to evaluate SARS-CoV-2 RdRp activity as well as potential inhibitors (Lu et al., 2020). They have shown that adding some remdesivir-TP to the reaction mix triggered chain termination. In addition, they have reported that 2’-C-methyl-GTP can be easily integrated into RNA. 2’-C-methyl-GTP derivative demonstrates efficacy to treat SARS-CoV-2 infection (Lu et al., 2020). 

Remdesivir is currently one of the FDA-approved drugs for COVID-19 treatment. Kokic et al. (2021) showed that Remdesivir’s active form operates as a nucleoside analog to inhibit SARS-CoV-2 RdRp (Kokic et al., 2021). Remdesivir is incorporated into the developing RNA product by the RdRp, which is allowing three additional nucleotides to be added before RNA synthesis stops. The structural analysis of the remdesivir-stalled state may hinder proofreading by the viral 3-exonuclease, suggesting a potent mode of inhibition by next-generation therapeutics targeting SARS-CoV-2 RdRp. Ribavirin is a ribonucleoside analog with a broad range of antiviral activity. Ribavirin antiviral mechanisms are debated and it is stated that it disrupts the viral RNA replication in the host or it can increase the immune response. A study showed that Ribavirin at a concentration of 109.50 μM and favipiravir at 61.88 μM caused 50% CPE in Vero cells infected with SARS-CoV-2 (Wang et al., 2020). Favipiravir was also used in the fight against COVID-19. Favipiravir reduces SARS-CoV-2 RNA replication (Naydenova, et al. 2021). The structure analysis indicates that favipiravir binds to the catalytic location of SARS-CoV-2 RdRp with a low rate of incorporation into the RNA primer strand (Naydenova et al., 2021). 

Sofosbuvir (GS‐7977; formerly PSI‐7977) is an FDA-approved hepatitis C virus (HCV) inhibitor, which is developed by Gilead (Rodriguez-Torres et al., 2013). This drug has been shown as the first to be used safely and to be effective with no need for the usage of interferon. The sofosbuvir with velpatasvir is approved as EPCLUSA and tested in HCV (Greig, 2016). HCV RdRp shows structural homology and replication mechanism similar to SARS-CoV-2 RdRp. A study conducted with polymerase extension assays stated that sofosbuvir active triphosphate form inhibited SARS-CoV-2 RdRp (Chien et al., 2020). Similarly, the other FDA-approved antivirals, which are Alovudine as an HIV/AIDS drug and Tenofovir alafenamide as an HIV/hepatitis B drug were tested for the inhibition of SARS-CoV-2 RdRp (Chien et al., 2020) have been shown to block the SARS-CoV-2 polymerase extension. 

Galidesivir (BCX4430) is a synthetic adenosine analogue and it has been shown to inhibit viral RNA polymerase in various RNA viruses including SARS-CoV and Mers-CoV (Warren et al., 2014). The role of BCX4430 in the inhibition of viral polymerase is stated as a nonobligate RNA chain terminator (Warren et al., 2014). The inhibitory activity of BCX4430 against SARS‐CoV was stated as 57.7 μM EC50 value and CC50 > 296 μM in HeLa cells by using high‐content image assays (Warren, et al., 2014; Pruijssers and Denison, 2019). 

EIDD‐1931 is the ribonucleoside analog β‐D‐N4‐hydroxycytidine. Several studies demonstrated the inhibitory function against influenza virus, Ebola, and several CoVs with the broad range antiviral spectrum activity (Agostini et al., 2019; Sheahan et al., 2020). The inhibitory effect of NHC against SARS-CoV-2 has been shown as EC50 of 0.3 μM in Vero cells and EC50 of 0.08 μM in Calu‐3 cells (Sheahan et al., 2020). EIDD‐2801 is an orally bioavailable NHC prodrug. EIDD‐2801 showed enhanced pulmonary function and diminished viral load in mice infected with SARS‐CoV or MERS‐CoV (Sheahan et al., 2020).

Recently, the stable nitroxide TEMPOL has been reported with a strong inhibitory effect on the RdRp activity of SARS-CoV-2 (Maio et al., 2021). NSP12 RdRp has been found to cooperate with two iron-sulfur (Fe-S) metal cofactors in the site of zinc centers. TEMPOL oxides Fe-S clusters and inhibits SARS-CoV-2 RdRp through the direct interaction within Fe-S clusters in RdRp that causes their degradation. In addition, it has been stated that TEMPOL displayed robust antiviral activity in Vero E6 cells infected with SARS-CoV-2 (Maio et al., 2021). This study indicates the potentiality of TEMPOL and other alternative stable nitroxides for the development of therapeutics against SARS-CoV-2. 

These studies, overall, suggest the potential of nucleoside analogues as polymerase inhibitors for the development of COVID-19 therapeutics.

### 2.8. NSP9: SARS-CoV-2 RNA replicase

The NSP9 is thought to participate in viral replication and pathogenicity. The structure of SARS-CoV-2 NSP9 was defined with unique fold characteristics as well as the dimerization interface. NSP9 binds to RNA through this distinctive fold feature of coronaviruses harboring the GxxxG interaction motif (Littler et al., 2020). Thus, the role of NSP9 was determined in the RNA/DNA binding activity. SARS-CoV NSP9 is able to form a dimer at the GXXXG motif. This dimerization of NSP9 has been shown as a critical and required step for the SARS-CoV viral replication, however, it is not necessary for RNA binding. In addition, in silico studies suggest that SARS-CoV-2 NSP9 could be targeted as shown with the identification of a potent inhibition pocket (Farias et al., 2021). In addition, known antivirals have been proposed to interact with this pocket and could decrease the flexibility of the monomer compared to the dimer. However, there are currently no known or validated small molecule inhibitors targeted to SARS-CoV-2 NSP9 protein.

### 2.9. NSP10 and NSP16: SARS-CoV-2’s 2′-O-ribose methyltransferase complex

The eukaryotic mRNAs have unique features with a 5’ methylated cap structure that is needed for mRNA stability and translation process. All viruses utilize host cell ribosomes for their protein translation and CoVs have been equipped with such a cap structure for their viral RNA. It has been demonstrated that cap structure with ribose 2′-O-methylation provides a viral escape from the recognition of the host’s innate immune system (Schoggins and Rice, 2011). SARS-CoVs encode the 2′-O-methyltransferase and it consists of two subunits which are the NSP16 catalytic subunit and NSP10 stimulatory subunit (Chen et al., 2011). This 2′-O-MTase activity is unique for CoVs compared to other viruses or hosts (Chen et al., 2011). It has been shown that SARS-CoV NSP10 is required for the NSP16 enzyme activity (Chen et al., 2011). NSP10 stimulates NSP16 to attach capped RNA and the methyl donor S-adenosyl-L-methionine (SAM) (Chen, et al. 2011). 

The function of the NSP10/16 complex is to catalyze the methylation of the RNA cap of the virus at the ribose 2′-O position. By methylation, the viral RNA cap-0 structure which is featured as 7MeGpppA… converted into 7MeGpppA2′-O-Me… cap-1 structure. This provides an advantage for mimicking cellular mRNAs and hence tricking the host immunity from viral RNAs recognition (Chen et al., 2011; Decroly et al., 2011). 

The SARS-CoV-2 NSP10/NSP16 2′-O-MTase has been characterized at the atomic level. It has been stated that SAM analogs SIN could target the conserved SAM binding pocket within the NSP10/NSP16 complex (Lin, et al. 2020). Studies suggest that the SARS-CoV-2 NSP10-NSP16 complex is druggable as much as other methyltransferases (reviewed in (Chang and Chen, 2021)). However, there is an unmet demand for an improved assay for small molecule screening. 

Yazi et al (2021) have recently reported a radioactivity-based assay for the methyltransferase activity of the NSP10-NSP16 complex, which is suitable for a 384-well plate (Khalili Yazdi et al., 2021). In addition, Perveen et al. (2021) reported a RNA displacement assay to target and analyze the activity of the SARS-CoV-2 NSP10-NSP16 complex (Perveen et al., 2021). They confirmed their assay with the purified NSP10-NSP16 complex with binding to the methyl donor SAM. In addition, they were able to observe the reaction product S-adenosyl-l-homocysteine (SAH) and showed inhibition of enzymatic assay with a common methyltransferase inhibitor, sinefungin. This was followed by a screening of thousands of drug-like compounds to determine RNA-competitive inhibitors in the NSP10-NSP16 complex in the hopes of finding COVID-19 treatments. Although they have determined three compounds with concentration-dependent inhibition, they failed to show any binding when tested by isothermal titration calorimetry.

### 2.10. NSP11: SARS-CoV-2, a minor cleavage product

SARS-CoV-2 NSP11 is a minor product of cleavage that comes from the Mpro pp1a processing (Gadhave et al., 2020; Gadhave et al., 2021). Its whole sequence of amino acids is SADAQSFLNGFAV. There has been no description of an independent NSP11 function. However, a recent study highlighted that SARS-CoV-2 NSP11 protein is inherently disordered and forms a helical shape in appropriate conditions. In comparison to neutral and anionic lipids, it demonstrates a great affinity for the SDS micelles and is ready to undergo conformational transformation. Intriguingly, Gadhave et al., (2020) suggest that SARS-CoV-2 NSP11 can operate in the affinity and contact between the cytosolic host membranes (Gadhave et al., 2020; Gadhave et al., 2021). 

### 2.11. NSP13: SARS-CoV-2’s helicase

SARS-CoV-2 NSP13 is a helicase, thereby it uses ATP hydrolysis to unwind duplex DNA and RNA with a 5′single-stranded tail in the 5′–3′ direction (Reviewed in (Suryawanshi, et al. 2021)). SARS-CoV-2 NSP13 along with other proteins were also shown to inhibit interferon production and nuclear localization of IRF3 (Yuen, et al. 2020). In addition, SARS-CoV-2 NSP13 along with NSP1 inhibited caspase-1 activity and IL-1 production caused by the NLRP3 inflammasome (Kim, et al. 2021). 

White et al. (2020) studied SARS-CoV-2 NSP13 helicase and performed an in silico screening followed by in vitro validation of two-hit compounds (White et al., 2020). Their targeting strategy involved docking into the ATP-binding site of the NSP3 helicase homology model. Lumacaftor and Cepharanthine were among the 10 tested compounds that showed IC50 values of 0.3 and 0.4 mM against SARS-CoV-2 in ATPase assay, respectively. Intriguingly, Cepharanthine was reported as an inhibitor of SARS-CoV suggesting that it may also inhibit SARS-CoV-2 viral replication (Zhang et al., 2005). 

### 2.12. NSP14: SARS-CoV-2 3′ to 5′ exoribonuclease and (guanine-N7)-methyltransferase

SARS-CoV-2 NSP14 mainly has a 3′ to 5′ exoribonuclease activity that removes mismatches that occur during viral genome replication. It interacts with NSP7, 8, 10, and 12 (reviewed in (Suryawanshi et al., 2021)). SARS-CoV-2 NSP14 has also a (guanine-N7)-methyltransferase (N7-MTase) activity that prevents newly generated viral RNA from being degraded. The presence of this exoribonuclease domain is required for its capping function, although the domain’s enzymatic activity was shown to be not necessary.

Renata et al. (2021) used a Py-FLINT assay to test about 7000 compounds for inhibition of SARS-CoV-2 NSP14 N7-methyltransferase (Kasprzyk et al., 2021). Pyridostatin, which was among the 33 most potent NSP14 N7-methyltransferase inhibitors, had an IC50 value of 3.19 μM and reduced viral replication with an EC50 of 3.58 μM and selectivity index of 16.6. Other NSP14 N7-MTase inhibitors Gastrodenol, theaflavin-3,3’-di-O-gallate, Reactive Blue 2, and Evans Blue demonstrated antiviral activity with IC50 values of 40.1, 33.2, 16.3, and 30.9 μM, respectively. In addition, Canal et al. (2021) tested about five thousand compounds and determined patulin and aurintricarboxylic acid nsp14 exoribonuclease inhibitors with antiviral activity in a Vero infection model (Canal, et al. 2021).

Basier et al. (2021) tested over 5000 compounds in vitro by homologous time-resolved fluorescence (HTRF) assay and reported four potent SARS-CoV-2 NSP14 RNA cap methyltransferase inhibitors with an antiviral activity based on the SARS-CoV-2 infection model (Basier, et al. 2021). PF-03882845, trifluperidol, inauhzin, lomeguatrib, and inhibited SARS-CoV-2 NSP14 with HTRF50 values of 1.1, 12.9, 23.0, and 53.8 μM, respectively. PF-03882845, inauhzin, and trifluperidol demonstrated antiviral EC50 of 10.97, 14.9, and 12.96 μM respectively. Lomeguatrib had EC50 about 60 μM and showed cytotoxicity. In addition, these compounds demonstrated synergy when combined with remdesivir to lower IC50 against SARS-CoV-2 in vitro. 

These findings suggest that targeting SARS-CoV-2 NSP14 is a plausible strategy in the development of sensible COVID-19 treatments.

### 2.13. NSP15: SARS-CoV-2 endoribonuclease

SARS-CoV-2 NSP15 demonstrates a uridine-specific and Mn(2+)-dependent endoribonuclease activity that reduces viral dsRNA sensing by host dsRNA sensors (Kim et al., 2020). Because of the structural likenesses, inhibitors of SARS-CoV NSP15 have a strong possibility of inhibiting the SARS-CoV-2 NSP15. Kim et al. (2021) recently developed a synthetic oligonucleotide substrate cleaved by SARS-CoV-2 NSP15 that allowed to identify tipiracil as a potent inhibitor of SARS-CoV-2 NSP15 endoribonuclease activity by binding to uridine binding site (Kim et al., 2020; Kim, et al., 2021). This is associated with a 50% reduction at 7.5 μM tipiracil treatment albeit with modest antiviral activity (EC50 of >50 μM) in vitro. 

## 3. Conclusions and discussion

This study addressed the validated inhibitors that target SARS-CoV-2 NPSs and their antiviral efficacy as a possible COVID-19 candidate therapeutic. Pharmaceutical repurposing and design of novel chemical entities in need to treat COIVID-19 play important roles in the rapid identification of effective medicines. As a result, resolving 3D structures as well as SARS-CoV-2 NSP specific enzymatic tests are often required for the discovery of COVID-19 specific inhibitors. 

The ideal inhibitors should have high specificity for the target and be effective as an antiviral in vitro as well as in vivo after being administered to SARS-CoV-2 animal models. This also requires inhibitors to possess a reasonable plasma half-life. Although many inhibitors described so far by in silico studies have high binding affinity to studied SARS-CoV-2 NSPs, they often lack validation by in vitro SARS-CoV-2 NSP specific enzymatic assays or lack antiviral activity. Thus, we highlighted only and focused on the validated inhibitors of SARS-CoV-2 NSPs in relation to their pharmaceutical mechanism of action (Table). In addition, we have summarized if they had any antiviral activity, in vivo efficacy against SARS-CoV-2 infections as well as their bioavailability and pharmacokinetics if the information is provided. 

Studies on SARS-CoV-2 biology concerning NSP complexes show that the majority of NSPs could be targeted by small molecules and they are druggable. For many of the NSPs, there have been reports or attempts to develop assays that could allow the screening of a large number of small molecules. SARS-CoV-2 NSP3, NSP5, NSP12, NSP13, and NSP15 have been shown to be inhibited at least by one small molecule in vitro in respective enzymatic assays as well as in vitro antiviral assays (Table). In addition, several inhibitors targeting SARS-CoV-2 NSP5 Mpro have been shown to be effective in vivo in SARS-CoV-2 infection models. Inhibitors of NSP3 or NSP5 proteases might possibly decrease the activity of important human proteases since viral proteases and human host proteases have comparable substrates and hydrolysis processes. Thus, it is critical to examine the selectivity of inhibitors to NSP3 or NSP5 proteases during hit selection, lead optimization, and drug development to assure specificity and selectivity of the final medicines against SARS-CoV-2 NSPs.

**Table:** Current status of validated small molecule inhibitors of SARS-CoV-2 NSPs.

SARS-CoV-2 protein	Validated inhibitors with IC50 values based on the in vitro enzymatic assays	Validated inhibitors with EC50 values based on the in vitro SARS-CoV-2 antiviral assays or in vivo efficacy	References
NSP1	Not reported	Not reported	-
NSP2	Not reported	Not reported	-
NSP3	Rac3j (1.4 μM), rac3k (1.15 μM), rac5c (0.81 μM), Levothyroxine (15.3. μM), manidipine-2HCl (14.2 μM), maprotiline (9.7 μM), reserpine (5.7 μM), loperamide (33.5 μM), proanthocyanidin (2.4 μM), GRL-0617 and its derivatives 4 and 7 (2.3–32.8 μM), Hypericin (90% inhibition at 100 μM).	Loperamide (11.4 μM), manidipine-2HCl (14.5 μM), maprotiline (9.3 μM),Levothyroxine (7.0 μM), proanthocyanidin (2.5 μM), and reserpine (6.6 μM), GRL-0617 and its derivatives 4 and 7 (1.4 to 5.2 μM).	(Klemm et al., 2020; Kuo et al., 2021; Osipiuk et al., 2021; Pitsillou et al., 2020)
NSP4	Not reported	Not reported	-
NSP5	11a (0.053 μM), 11b (0.040 μM), 11r (0.18 μM), 13a (2.39 μM), 13b (0.67μM), Thimerosal (0.6 μM), phenylmercuric acetate (0.4 μM), Evans blue (0.2 μM), Tannic acid (2.1 μM), Hematoporphyrin (3.9 μM), baicalin (6.41 μM), baicalein (0.94 μM), rottlerin (37 μM), M-8524 (CID46897844, 31 μM), MI-09 (0.015 μM), MI-30 (0.017 μM), compound 4 (0.15 μM), GC376 (0.16 μM), MAC-5576 (0.08 μM), Cinanserin (125 μM), N3 (N.A.), Disulfiram (9.35 μM), Ebselen (0.67 μM), Carmofur (1.82 μM), Tideglusib (1.55 μM), PX-12 (21.39 μM), Shikonin (15.75 μM).	11a (0.53 μM), 11b (0.72 μM), 13b (4–5 μM), baicalin (10.27 μM), baicalein (1.69 μM), MI-09 (0.86–1.2 μM in vitro and in vivo antiviral efficacy), MI-30 (0.54–1.1 μM in vitro and in vivo antiviral efficacy), compound 4 (2.88 μM), GC376 (2.19 μM), Cinanserin (20.6 μM), N3 (16.77 μM), Ebselen (4.67 μM)	(Coelho et al., 2020; Dai et al., 2020; Glaab et al., 2021; Iketani et al., 2021; Jin et al., 2020; Qiao et al., 2021; Su et al., 2020; Vuong et al., 2021; Zhang et al., 2020)
NSP6	Not reported	Not reported	-
NSP7	Not reported (see NPS12)	Not reported (see NPS12)	-
NSP8	Not reported (see NPS12)	Not reported (see NPS12)	-
NSP9	Not reported	Not reported	-
NSP10	Not reported	Not reported	-
NSP11	Not reported	Not reported	-
NSP12	NHC, Remdesivir, Favipiravir, 2’-C-methyl-GTP, nitroxide TEMPOL, Sofosbuvir, Tenofovir alafenamide	NHC (0.08–0.3 μM), Remdesivir (109.5 μM), Favipiravir (61.8 μM), 2’-C-methyl-GTP derivative, nitroxide TEMPOL	(Chien et al., 2020; Lu et al., 2020; Maio et al., 2021; Sheahan et al., 2020) (Wang et al., 2020)
NSP13	Lumacaftor (0.3 mM) and Cepharanthine and (0.4 mM)	Cepharanthine was reported as an inhibitor of SARS-coV1. Not tested for SARS-CoV-2 viral replication yet.	(White et al., 2020)
NSP14	Pyridostatin (3.19 μM and selectivity index of 16.6), PF-03882845 (1.1 μM), Trifluperidol (12.9 μM), Inauhzin (23.0 μM), Lomeguatrib (53.8 μM), Gastrodenol (1.08 μM), theaflavin-3,3’-di-O-gallate (1.36 μM) , Reactive Blue 2 (4.12 μM), Evans Blue (4.84 μM), patulin (1.8 μM) and aurintricarboxylic acid (10.3 μM)	Pyridostatin (3.58 μM), PF-03882845 (10.97 μM), Inauhzin (14.9 μM), Trifluperidol (12.96 μM), Lomeguatrib (~ 60 μM with cytotoxicity), Reactive Blue 2 (16.3 μM) Gastrodenol (40.1 μM), theaflavin-3,3’-di-O-gallate (33.2 μM), Evans Blue (30.9 μM).	(Basier et al., 2021; Canal et al., 2021; Kasprzyk et al., 2021)
NSP15	Tipiracil (~ 7.5 μM)	Tipiracil (> 50 μM)	(Kim et al., 2021)
NSP16	Not reported	Not reported	-

In summary, a tremendous effort done by scientists globally in less than a year showed that several viral NSPs are druggable with validated small molecules that are efficacious in vitro, in vivo, and with a reasonable plasma half-life. Further studies in drug development soon could allow the generation of COVID-19 specific medicines by blocking key enzymes involved in pathogenesis, replication, and infection of SARS-CoV-2. However, more research is needed to develop specific inhibitors for the majority of NSPs.

****
